# Effect of IV alteplase on the ischemic brain lesion at 24–48 hours after ischemic stroke

**DOI:** 10.1212/WNL.0000000000006575

**Published:** 2018-11-27

**Authors:** Grant Mair, Rüdiger von Kummer, Zoe Morris, Anders von Heijne, Nick Bradey, Lesley Cala, André Peeters, Andrew J. Farrall, Alessandro Adami, Gillian Potter, Peter A.G. Sandercock, Richard I. Lindley, Joanna M. Wardlaw, for the IST-3 Collaborative Group

**Affiliations:** From Edinburgh Imaging, and UK Dementia Research Institute at the University of Edinburgh and Centre for Clinical Brain Sciences (G.M., Z.M., A.J.F., J.M.W.), and Division of Clinical Neurosciences (P.A.G.S.), University of Edinburgh, UK; Department of Neuroradiology (R.v.K.), Dresden University Stroke Centre, Germany; Danderyd Hospital (A.v.H.), Stockholm, Sweden; Neuroradiology (N.B.), James Cook University Hospital, Middlesborough, UK; School of Medicine (L.C.), University of Western Australia; Cliniques Universitaires St Luc (A.P.), Neurologie, Belgium; Stroke Center (A.A.), Department of Neurology, IRCCS Sacro Cuore Don Calabria Hospital, Negrar, Verona, Italy; Department of Neuroradiology (G.P.), Salford Royal NHS Foundation Trust, Manchester, UK; and Westmead Hospital Clinical School and The George Institute for Global Health (R.I.L.), University of Sydney, Australia.

## Abstract

**Objective:**

To determine whether alteplase alters the development of ischemic lesions on brain imaging after stroke.

**Methods:**

The Third International Stroke Trial (IST-3) was a randomized controlled trial of IV alteplase for ischemic stroke. We assessed CT or brain MRI at baseline (pretreatment) and 24 to 48 hours posttreatment for acute lesion visibility, extent, and swelling, masked to all other data. We analyzed associations between treatment allocation, change in brain tissue appearances between baseline and follow-up imaging, and 6-month functional outcome in IST-3. We performed a meta-analysis of randomized trials of alteplase vs control with pre- and postrandomization imaging.

**Results:**

Of 3,035 patients recruited in IST-3, 2,916 had baseline and follow-up brain imaging. Progression in either lesion extent or swelling independently predicted poorer 6-month outcome (adjusted odds ratio [OR] = 0.92, 95% confidence interval [CI] 0.88–0.96, *p* < 0.001; OR = 0.73, 95% CI 0.66–0.79, *p* < 0.001, respectively). Patients allocated alteplase were less likely than controls to develop increased lesion visibility at follow-up (OR = 0.77, 95% CI 0.67–0.89, *p* < 0.001), but there was no evidence that alteplase reduced progression of lesion extent or swelling. In meta-analysis of 6 trials including IST-3 (n = 4,757), allocation to alteplase was associated with a reduction in ischemic lesion extent on follow-up imaging (OR = 0.85, 95% CI 0.76–0.95, *p* = 0.004).

**Conclusion:**

Alteplase was associated with reduced short-term progression in lesion visibility. In meta-analysis, alteplase reduced lesion extent. These findings may indicate that alteplase improves functional outcome by reducing tissue damage.

**Classification of evidence:**

This study provides Class II evidence that IV alteplase impedes the progression of ischemic brain lesions on imaging after stroke.

In patients presenting acutely with ischemic stroke, brain imaging can demonstrate ischemia and infarction based on water shifts within brain tissue.^1^ On CT, brain tissue becomes gradually more hypoattenuated as its water content increases.^2^ Within the first few hours after stroke onset, there is a reduction in gray matter attenuation so that gray matter becomes of similar attenuation to normal white matter resulting in the well-described loss of visibility of the insular ribbon, basal ganglia, or affected cortex.^3,4^ These early attenuation changes of the brain are often not apparent immediately after symptom onset, but by 24 hours, affected brain is usually obviously hypoattenuated compared with normal brain,^5^ and is specific for infarction.^6^ On MRI, diffusion-weighted imaging (DWI) can show hyperintensity in ischemic tissue within minutes of stroke onset. Hours later, the lesion also becomes hyperintense on other T2-weighted sequences^7^ and, similar to hypoattenuation on CT, this later change usually indicates infarction.^8^ Both CT and MRI show brain tissue swelling as a secondary indicator of injury.

Treatment with IV alteplase within the first few hours after stroke improves long-term clinical outcome and accelerates disappearance of arterial obstruction.^9–11^ However, it is unclear whether IV alteplase modifies progression of the appearance of acutely affected brain tissue on imaging (i.e., the ischemic lesion) over the first 24 to 48 hours after stroke, or whether any short-term alteplase-related alteration in lesion progression might explain the long-term improvement in functional outcome.

The Third International Stroke Trial (IST-3) was a large multicenter, randomized controlled trial testing IV alteplase given within 6 hours of ischemic stroke.^12^ In the present analysis, our aim was to assess the effect of alteplase on any change in ischemic lesion appearance on CT or MRI between pretreatment (baseline) and 24- to 48-hour posttreatment follow-up imaging. We investigated whether alteplase modified short-term progression of CT hypoattenuation or magnetic resonance T2-weighted hyperintensity (lesion visibility), lesion extent, or tissue swelling compared to control and tested whether alteration in short-term progression of the lesion appearance predicted long-term functional outcome after ischemic stroke. We set our results in the context of a meta-analysis of all available data from randomized controlled trials.

## Methods

### Standard protocol approvals, registrations, and patient consents

IST-3 was an international, multicenter PROBE (prospective, randomized, open-label, blinded endpoint) trial of IV alteplase (recombinant tissue plasminogen activator) for ischemic stroke.^12^ Ethical approval was granted by the Scotland A research ethics committee and by local ethics committees. IST-3 was registered with Current Controlled Trials (ISRCTN25765518). Enrollment, data collection, and CONSORT (Consolidated Standards of Reporting Trials) compliance have been described.^12,13^

Briefly, adult patients with acute stroke of any severity (assessed with the NIH Stroke Scale [NIHSS]), with no upper age limit, were eligible if treatment could be started within 6 hours of symptom onset and brain imaging had excluded intracranial hemorrhage and structural stroke mimics. Informed consent for research was obtained for all patients. The full trial protocol is available: dcn.ed.ac.uk/ist3/. IST-3 primary results are published.^12^

Patients were randomly allocated to IV alteplase (0.9 mg/kg) or control. Treating clinicians used an automated telephone or online system to enter baseline data and obtain a randomized treatment allocation. Excepting the first 276 patients (double-blind phase), treatment was given open label. Patients were followed up at 6 months by postal or telephone questionnaire to assess functional status with the Oxford Handicap Scale^14^ by assessors who were masked to clinical and imaging findings and treatment allocation.

### Brain imaging

The IST-3 imaging protocol has been described^4,12,15^: CT or MRI was required pretreatment at baseline and follow-up. For CT, the maximum slice thickness was 5 mm through the posterior fossa and 10 mm for the cerebrum, but most CT was performed with thinner slices. MRI included T1, T2, DWI, fluid-attenuated inversion recovery, and T2*. DICOM (Digital Imaging and Communications in Medicine) was collated centrally and anonymized. We excluded patients from the present analysis if images were not received centrally.

### Image analysis

A panel of 10 experienced neuroradiologists and neurologists assessed imaging with a secure online viewing tool, which was developed after extensive review of early ischemic signs^3^ and observer reliability testing.^4,16,17^ The Systematic Image Review System includes a validated data collection pro forma (available at ed.ac.uk/edinburgh-imaging/image-analysis-tools), which underwent extensive observer reliability testing^16,17^ to ensure there was satisfactory agreement (κ >0.7) between readers for all imaging features assessed.^4^ Image assessors were masked to clinical data and treatment allocation. We scored baseline imaging (prerandomization) separately and blindly to follow-up imaging (24–48 hours after stroke onset) and vice versa, although assessors were aware if imaging was acquired at baseline or follow-up.

We reviewed all baseline and follow-up imaging for evidence of acute ischemia/recent infarct (i.e., the acute lesion) using the validated visual scores to assess 3 features: (1) acute lesion visibility; (2) location and extent of the lesion; and (3) lesion swelling.

#### Acute lesion visibility

**Figure 1:**
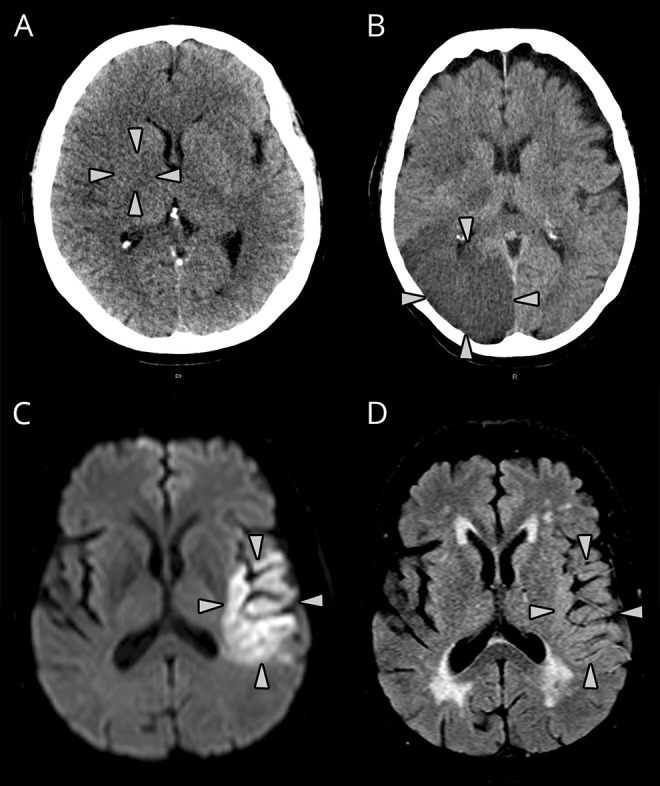
CT and MRI examples of grading of acute ischemic lesion visibility (A) CT lesion visibility grade 1; attenuation of affected gray matter (right lentiform nucleus, arrows) equivalent to normal surrounding white matter. (B) CT lesion visibility grade 2; attenuation of affected right occipital lobe (gray and white matter, arrows) less than normal white matter. (C) Diffusion-weighted and (D) fluid-attenuated inversion recovery (T2-based) imaging demonstrating MRI lesion visibility grade 1; acute lesion is clearly visible in panel C (arrows) but only faintly visible in panel D (arrows).

We graded acute lesion visibility on a 3-point ordinal scale to reflect the range of progressive tissue changes seen after ischemic stroke, i.e., increasingly more visible CT hypoattenuation or MRI T2-weighted hyperintensity (e.g., figure 1). This schema reflects well-described tissue changes^3,4,16,17^:

- On CT, we defined acute lesion visibility as grade 0 (no acute lesion visible); subtle, or grade 1 (gray matter attenuation equal to that of normal white matter); and severe, or grade 2 (gray and/or white matter attenuation lower than normal white matter).
- On MRI, we defined lesions as grade 0 (no lesion); subtle, or grade 1 (hyperintense area on DWI but not on other T2-weighted sequences); and severe or grade 2 (hyperintense on T2-weighted sequences with or without DWI hyperintensity).^4^

#### Location and extent of acute lesion

We used the IST-3 Ischemic Lesion Score and ASPECTS (Alberta Stroke Program Early CT Score) to assess the location and extent of acute lesions.^18,19^

The IST-3 Ischemic Lesion Score records the lesion extent according to common patterns of infarction in all major vascular territories (anterior, middle [MCA], and posterior cerebral arteries), the brainstem, or cerebellum, each with subdivisions. It includes an 8-point scale for the MCA territory.^4,12,18^ We condensed the detailed scores into fewer groups for analysis, as described previously^4,12^: 0 = no acute lesion; 1 = small (e.g., lacunar or small cortical lesion); 2 = medium (e.g., striatocapsular lesion or superficial MCA territory); 3 = large (e.g., complete MCA territory); and 4 = very large (e.g., complete MCA plus anterior cerebral artery territories). See data available from Edinburgh DataShare (figure e-1, dx.doi.org/10.7488/ds/2367).

ASPECTS assesses lesion extent only in the MCA territory, in 10 sections, scoring a point for each area that is affected, and ranges from 10 = normal (no MCA territory lesion) to 0 = acute lesion affecting the entire MCA territory.^19^

#### Acute lesion swelling

We graded tissue swelling on a validated 7-point scale based on sulcal or ventricular effacement or midline shift, from none (0) to severe (6 = midline shift with effacement of the basal cisterns).^18^ See data available from Edinburgh DataShare (figure e-2, dx.doi.org/10.7488/ds/2367).

We also assessed baseline scans for leukoaraiosis, atrophy, and old stroke lesions (i.e., prestroke signs) using validated scores.^4,20–22^ We noted the presence of any hemorrhage at follow-up.

### Data analysis

We compared baseline and follow-up imaging to detect change in any of the acute ischemic lesion appearances. We subtracted the baseline imaging scores from the follow-up imaging scores. For the variables lesion visibility grade, IST-3 Ischemic Lesion Score, and swelling, a positive value represented imaging progression. For ASPECTS, a negative value represented imaging progression.

We used univariate tests to compare the alteplase and control groups and to assess for associations with imaging progression. We then used multivariable ordinal regression to identify predictors of imaging progression (in those with a significant univariate association, i.e., change in lesion visibility grade) and 6-month functional outcome. Finally, we tested for interactions with alteplase between subgroups of variables predictive of imaging progression (*p* < 0.1) on ordinal regression analysis. Because of the risk of confounding, we did not include ASPECTS and IST-3 Ischemic Lesion Score in the same multivariable models. We adjusted regression analyses for the key outcome predictors of age, NIHSS, time between baseline and follow-up scans, and presence of hemorrhage at follow-up, plus leukoaraiosis, atrophy, and old stroke lesions since we showed previously in IST-3 and other datasets that these prestroke signs affect lesion visibility^16,17^ and are adverse prognostic markers^4,12^ (therefore, we did not repeat these tests in the present analysis). To stabilize regression estimates, we grouped the variable *time between scans* into 6 time windows (0–11, 12–23, 24–35, 36–47, 48–59, 60+ hours) and we combined the 3 most severe grades of the Oxford Handicap Scale into a single category, resulting in 5 functional outcome grades (0, 1, 2, 3, 4–6).

We used IBM SPSS Statistics software, version 21.0 (IBM Corp., Armonk, NY) for all analyses unless otherwise stated, and considered *p* < 0.05 significant.

### Meta-analysis

We used the 2014 Cochrane systematic review *Thrombolysis for Acute Ischemic Stroke*^23^ to identify randomized controlled trials of IV alteplase that reported outcomes by imaging assessment of ischemic lesions at baseline and follow-up. Specifically, we sought comparable data for the 3 imaging features examined in IST-3: namely, acute lesion visibility; lesion extent (scored as *lesion volume* if assessed at only one time point, and *lesion growth* if assessed at more than one time point); and lesion swelling. In addition, for each relevant trial identified, we searched PubMed for any post hoc or subgroup analyses published up to the end of January 2018.

We used Comprehensive Meta-Analysis software, version 2 (Biostat, Englewood, NJ) to compute odds ratios (ORs) of the alteplase effect for each trial dataset and to calculate summary statistics using a random effects model. We used *I*^2^ statistics to assess heterogeneity between studies.

### Data availability

IST-3 data are available on request to bona fide researchers via Edinburgh DataShare (datashare.is.ed.ac.uk/handle/10283/1931).

### Classification of evidence

Our primary objective was to determine whether alteplase alters the development of ischemic lesions on brain imaging after stroke. This study provides Class II evidence that IV alteplase (0.9 mg/kg) impedes the development of ischemic brain lesions according to 2 distinct imaging features. In IST-3, alteplase reduced short-term progression in lesion visibility (adjusted OR = 0.77, 95% confidence interval [CI] 0.67–0.89), and when combined in meta-analysis with all available randomized controlled trial data, alteplase reduced the extent of the ischemic brain lesion on follow-up imaging (adjusted OR = 0.85, 95% CI 0.76–0.95).

## Results

IST-3 recruited 3,035 patients. Baseline or follow-up imaging was not available for central review in 18 (0.6%) and 105 patients (3.5%), respectively. Central imaging review did not occur if the patient had died or was too unwell at follow-up, or if completed scans were never received centrally or were corrupted. Thus, expert-reviewed baseline and follow-up imaging was available for 2,916 patients (96.1%) (data available from Edinburgh DataShare, figure e-3, dx.doi.org/10.7488/ds/2367). Most had noncontrast CT performed at baseline (2,861, 98.1%) and at follow-up (2,766, 94.9%). MRI was used in 55 and 150 patients, respectively.

For the 2,916 patients included in this analysis, 1,416 (48.6%) were male, median age was 81 years (interquartile range [IQR] 72–86 years), and the median baseline NIHSS score was 11 (IQR 6–17). Median time from stroke onset to baseline scan was 154 minutes (IQR 105–215 minutes) while the median time between baseline and follow-up scans was 26 hours (IQR 24–36 hours). Treatment allocation was 1,474 patients (50.5%) to alteplase and 1,442 (49.5%) to control. We found no differences in demographic or clinical characteristics between the 2,916 patients with complete imaging data in these analyses and the 3,035 patients in the whole IST-3 trial (data not shown).

Among the 2,916 patients with complete imaging data, baseline demographic, clinical, and imaging variables were not different between treatment groups (table 1), except that patients in the control group had a slightly longer time lapse between baseline and follow-up imaging (IQR 24–40 vs 23–30 hours in the group allocated to alteplase, *p* < 0.001).

**Table 1:**
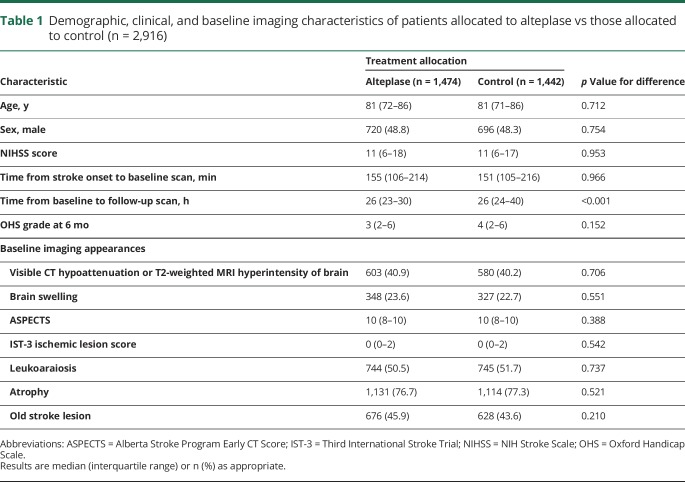
Demographic, clinical, and baseline imaging characteristics of patients allocated to alteplase vs those allocated to control (n = 2,916)

### Change in acute lesion between baseline and follow-up in IST-3

We identified an acute lesion (any of reduced tissue CT attenuation/increased T2-weighted hyperintensity, or swelling) in 1,183 patients at baseline and 2,124 patients at follow-up. Most acute lesions involved the MCA territory (baseline 1,088/1,178, 92.4%, follow-up 1,722/2,119, 81.3%). We identified any hemorrhage on 479/2,916 (16.4%) follow-up scans (table 2).

**Table 2:**
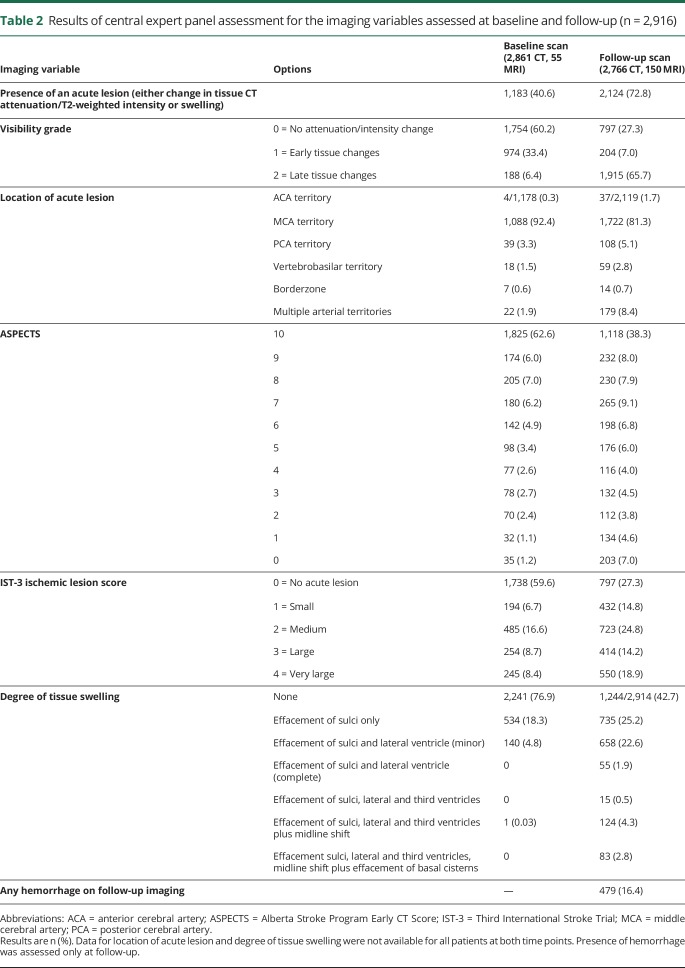
Results of central expert panel assessment for the imaging variables assessed at baseline and follow-up (n = 2,916)

Lesion visibility grade changed between baseline and follow-up in 1,994/2,916 patients (68.4%). Most patients had no visible lesion at baseline (1,754/2,916, 60.2%); in contrast, 2,119/2,916 (72.7%) had a visible lesion at follow-up (table 2). The prevalence of visible ischemic lesions at baseline did not differ between patients who presented early (0–3 hours from symptom onset [40.4%]) vs later (4–6 hours [40.8%]) (χ^2^ = 0.04, *p* = 0.834). Some lesions completely disappeared (76/2,916, 2.6%) between baseline and follow-up, fewer became less visible but remained present (11/2,916, 0.4%). Overall, lesion visibility increased in fewer patients allocated to alteplase (936/1,474, 63.5%) than to control (971/1,442, 67.3%), and lesion visibility decreased in more patients allocated to alteplase (46/1,474, 3.1%) than to control (41/1,442, 2.8%) (*p* = 0.007) (table 3).

**Table 3:**
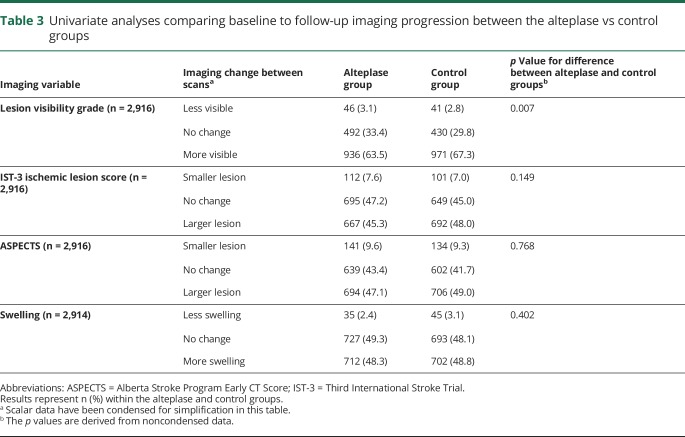
Univariate analyses comparing baseline to follow-up imaging progression between the alteplase vs control groups

Acute lesion extent increased in 1,344/2,916 (46.1%) on the IST-3 Ischemic Lesion Score and 1,241/2,916 (42.6%) on ASPECTS among all patients. Although there were slightly fewer patients with lesion growth and more with a reduction in acute lesion extent between baseline and follow-up (on either IST-3 Ischemic Lesion Score or ASPECTS) among those allocated to alteplase vs those allocated to control, these changes were not significant in IST-3 (tables 2 and 3).

Swelling changed (mostly increased) between baseline and follow-up in 1,494/2,914 patients (51.3%) overall (table 2). However, there was no difference in the change in swelling from baseline to follow-up scanning between treatment groups (table 3).

On ordinal regression analysis, compared with control, patients allocated to alteplase were less likely to show an increase in acute lesion visibility grade between baseline and follow-up scans (OR = 0.77, 95% CI 0.67–0.89, *p* < 0.001) (table 4). Leukoaraiosis on baseline imaging independently predicted a less visible lesion on follow-up imaging (OR = 0.78, 95% CI 0.67–0.91, *p* = 0.002), but patients with leukoaraiosis were less likely to have a visible lesion both at baseline (562/1,489, 37.7% with leukoaraiosis vs 621/1,427, 43.5% without leukoaraiosis, χ^2^ = 10.1, *p* = 0.002) and follow-up (1,034/1,489, 69.4% with leukoaraiosis vs 1,090/1,427, 76.4% without, χ^2^ = 17.7, *p* < 0.001). The following independently predicted development of a more visible lesion at follow-up: higher NIHSS score at randomization (OR = 1.06, 95% CI 1.05–1.07, *p* < 0.001); the presence of an old stroke lesion at baseline (OR = 1.19, 95% CI 1.03–1.37, *p* = 0.017); or hemorrhage on follow-up imaging (OR = 1.62, 95% CI 1.34–1.96, *p* < 0.001). Our results did not change if patients with MRI (at either baseline or follow-up) were excluded: in those with CT at both time points, OR for the effect of treatment on change in lesion visibility grade = 0.76 (95% CI 0.66–0.88, *p* < 0.001, n = 2,731; full data not shown).

**Table 4:**
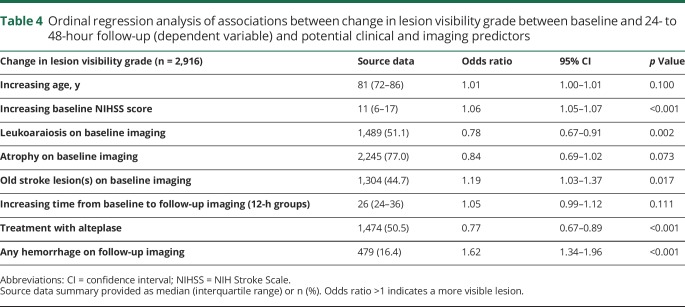
Ordinal regression analysis of associations between change in lesion visibility grade between baseline and 24- to 48-hour follow-up (dependent variable) and potential clinical and imaging predictors

We found no evidence of an interaction between alteplase and prespecified subgroups on change in acute lesion visibility grade between baseline and follow-up (ordinal regression analysis): baseline stroke severity mild (NIHSS score <8) vs moderate-severe (NIHSS score ≥8) (*p* = 0.842); presence vs absence of leukoaraiosis, atrophy, or old stroke lesions (*p* = 0.871, *p* = 0.358, *p* = 0.239, respectively) (data available from Edinburgh DataShare, figure e-4, dx.doi.org/10.7488/ds/2367).

### Effect of imaging appearances and clinical features on 6-month outcome in IST-3

On univariate analysis, the change in IST-3 Ischemic Lesion Score (*r* = 0.21, *p* < 0.001), ASPECTS (*r* = −0.35, *p* < 0.001), acute lesion visibility grade (*r* = 0.22, *p* < 0.001), and swelling (*r* = 0.40, *p* < 0.001) between baseline and follow-up imaging were all associated with 6-month functional outcome such that a worsening of imaging appearances between baseline and 24–48 hours was associated with worse 6-month functional outcome.

On ordinal regression analysis, the following were independent predictors of poor outcome: greater age (OR = 0.97, *p* < 0.001); higher baseline NIHSS score (OR = 0.86, *p* < 0.001); leukoaraiosis (OR = 0.72, *p* < 0.001) or atrophy (OR = 0.77, *p* = 0.013) on baseline imaging; increasing acute lesion extent (OR = 0.92, *p* < 0.001) or swelling (OR = 0.73, *p* < 0.001) between baseline and follow-up imaging; and hemorrhage on follow-up imaging (OR = 0.74, *p* = 0.017). Treatment with alteplase (OR = 1.36, *p* < 0.001) and increased time between baseline and follow-up imaging (OR = 1.10, *p* = 0.009) independently predicted better outcome at 6 months. Old stroke lesions and increasing visibility grade of the acute lesion between baseline and follow-up did not independently predict outcome (table 5). Results were similar if IST-3 Ischemic Lesion Score was used instead of ASPECTS to assess change in lesion extent (data not shown).

**Table 5:**
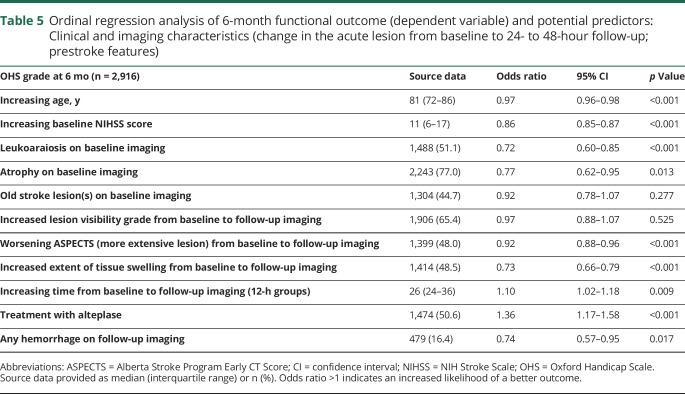
Ordinal regression analysis of 6-month functional outcome (dependent variable) and potential predictors: Clinical and imaging characteristics (change in the acute lesion from baseline to 24- to 48-hour follow-up; prestroke features)

### Meta-analysis

There have been no new randomized controlled trials of IV alteplase vs control since publication of the Cochrane review in 2014. The Cochrane review already contained a meta-analysis assessing the alteplase effect on symptomatic edema in 6 trials (including IST-3), which we did not repeat. Two of the trial datasets identified using Cochrane (ECASS [European Cooperative Acute Stroke Study], EPITHET [Echoplanar Imaging Thrombolytic Evaluation Trial])^24,25^ had examined lesion growth between baseline and follow-up similar to IST-3, while 3 of the trials (NINDS [National Institute of Neurological Disorders and Stroke], ATLANTIS [Alteplase Thrombolysis for Acute Noninterventional Therapy in Ischemic Stroke] A & B)^26–28^ had measured lesion volume on follow-up imaging alone (total n = 1,841).

**Figure 2:**
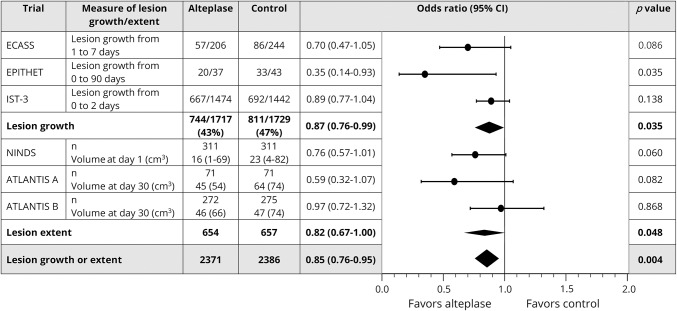
Meta-analysis of data from randomized controlled trials of alteplase that assessed short-term lesion growth or lesion extent on follow-up imaging For all studies in the combined analysis, *I*^2^ = 31.5%. ATLANTIS = Alteplase Thrombolysis for Acute Noninterventional Therapy in Ischemic Stroke; CI = confidence interval; ECASS = European Cooperative Acute Stroke Study; EPITHET = Echoplanar Imaging Thrombolytic Evaluation Trial; IST-3 = Third International Stroke Trial; NINDS = National Institute of Neurological Disorders and Stroke.

On meta-analysis, we found that alteplase impeded lesion growth (OR = 0.87, 95% CI 0.76–0.99) and was associated with smaller lesion volumes on follow-up imaging (OR = 0.82, 95% CI 0.67–1.00). When combined in a single meta-analysis including nearly 5,000 patients from 6 trials (including IST-3), alteplase reduced progression in the extent of the ischemic lesion (increased lesion growth or greater lesion volume on follow-up imaging) (OR = 0.85, 95% CI 0.76–0.95, *p* = 0.004) (figure 2).

## Discussion

In this large randomized trial with baseline and follow-up CT or MRI available for blinded central review in more than 97% of patients, we found that during the first 24 to 48 hours after the onset of ischemic stroke, patients allocated to alteplase were less likely to show progression in the visibility of the acute lesion compared with controls. Remarkably, less than 50% of patients in both treatment arms showed any lesion growth. IV alteplase did not reduce significantly the extent of the acute lesion or alter the degree of lesion swelling in IST-3, although there were fewer patients with larger lesions and more patients with smaller or stable lesions at 24 to 48 hours who received alteplase than control. It seems likely that IST-3 was underpowered to detect this subtle change in lesion extent, but when IST-3 data were combined in a meta-analysis of 6 randomized controlled trials of alteplase including nearly 5,000 patients, we found that alteplase was associated with a significant reduction in progression of the ischemic lesion extent. This alteplase effect includes a reduction in lesion growth between baseline and follow-up and a smaller lesion volume at follow-up. We postulate that IV alteplase resulted in fewer patients either converting an area of reversible ischemia to infarction, or expanding their ischemic lesion into tissue that was unaffected at baseline—both effects result in less injured brain tissue and may explain the better functional outcome seen following treatment with alteplase.

Background prestroke brain imaging features and baseline stroke severity were also independent predictors of change in acute lesion visibility from baseline to follow-up in IST-3. Patients with old infarcts were more likely to have increased acute lesion visibility at 24 to 48 hours. Perhaps individuals with prior infarcts are more likely to develop tissue injury if they experience another stroke than are patients without old infarcts on imaging, as suggested for lesion visibility on MRI in patients with minor stroke.^29^ Harder to explain is the finding that prestroke leukoaraiosis appeared to reduce progression of acute lesion visibility at follow-up. This may be because acute lesions were less often seen in patients with chronically abnormal white matter at baseline, a discrepancy that increased on follow-up imaging, but the reason for this is unclear.

Increased stroke severity (NIHSS) at baseline was a powerful independent predictor for increased lesion visibility on follow-up imaging in IST-3 in keeping with current and previously demonstrated associations between NIHSS, other imaging measures of severity (hyperattenuated arteries, arterial occlusion on angiography, acute lesion extent), and outcome after ischemic stroke.^10,11,30^ However, we found no evidence of an interaction between alteplase and severe vs mild stroke or presence/absence of prestroke features on change in acute lesion visibility; i.e., alteplase worked equally well across all of these subgroups.

Worsening of imaging appearances from baseline to 24–48 hours independently predicted poor outcome at 6 months in IST-3. Specifically, greater change in acute lesion extent on ASPECTS and increased tissue swelling were both associated with poor outcome. In addition, increased time from baseline to follow-up imaging was associated with better functional outcome, perhaps because of residual confounding with milder strokes being reimaged later. However, despite a significant univariate correlation between greater lesion visibility from baseline to 24- to 48-hour follow-up imaging and functional outcome, changes in lesion visibility did not independently predict outcome. This may reflect that increased lesion visibility in patients at follow-up in our analysis may include new infarcts appearing since baseline imaging (likely to affect functional outcome) and early infarcts at baseline becoming more visible secondary to the expected short-term tissue changes of infarct (less likely to affect functional outcome). Whether the transition from ischemic to infarcted brain can be reliably differentiated using unenhanced CT or basic MRI sequences remains unproven.

Lack of an association between alteplase and change in the degree of swelling at 24- to 48-hour follow-up appears to contrast with the significant excess of symptomatic brain swelling (that is, severe swelling on follow-up imaging plus neurologic deterioration within 7 days) among alteplase-treated patients in the primary IST-3 report.^12^ However, the current analysis examined all grades of swelling rather than only symptomatic swelling, and as noted above, progressive swelling was associated with worse functional outcome at 6 months.

The limitations of IST-3 have been discussed previously,^12^ chiefly the potential for bias because of the open trial design; however, for the present analyses, all readers were masked to clinical data and treatment allocation. In IST-3, investigators were required to perform follow-up imaging between 24 and 48 hours unless the patient deteriorated clinically, in which case immediate rescanning was required; patients who deteriorated and hence were scanned sooner were more likely to have poor 6-month outcomes. Patients treated with alteplase underwent follow-up imaging marginally sooner than controls but had better outcomes overall. It is likely that this marginal difference in scan interval may reflect the open design and the less-pressing need to reimage patients known to have been allocated to the control group. Although almost all patients had CT imaging at baseline and follow-up, a few patients were scanned with different modalities at the 2 time points. It may not be appropriate to compare acute lesion extent and visibility between CT and MRI, though it is perhaps reasonable to assume that classification of swelling would not differ between modalities. However, the results remained the same when those with MRI were excluded from the analysis assessing change in lesion visibility; we have therefore left all patients in for completeness. Validated quantitative computational methods for the assessment of brain imaging are not yet available for all of the characteristics we assessed in IST-3. Most important, computational lesion volume measurement does not distinguish between an increase in volume due to a true change in lesion extent and an increase in volume due to more swelling in a lesion of the same extent. We and others are developing computational lesion assessment methods,^31^ but their superiority over human visual rating remains unproven. Finally, angiographic imaging at baseline and follow-up was not available. We are aware that we do not know whether and to what extent treatment with alteplase was associated with arterial recanalization, and tissue reperfusion. The strengths of IST-3 include its large sample size, the inclusion of patients with a wide range of clinical characteristics and baseline imaging appearances, and central masked review of all imaging and visual scoring methods that had undergone extensive independent validation.^16,17^

We provide robust evidence that in ischemic stroke, allocation to alteplase was associated with less progression in the extent (based on the totality of the randomized evidence from the meta-analysis) and visibility (in IST-3 alone) of the acute lesion on short-term imaging follow-up. These findings may reflect less tissue damage among patients treated with alteplase and help explain how alteplase improves functional outcome after ischemic stroke. Alteplase did not alter the development of lesion swelling, but progression of lesion extent and swelling were both associated with poorer clinical outcome at 6 months. Imaging biomarkers of the effect of alteplase on ischemic brain will be valuable for clinical practice and research. Our findings indicate that early changes in the ischemic brain lesion are measurable on imaging, that these imaging appearances can predict outcome, and that treatment with alteplase limits progression of the ischemic lesion, which may in turn act as a surrogate for an improved functional outcome.

## Glossary

ASPECTS: Alberta Stroke Program Early CT Score
CI: confidence interval
DWI: diffusion-weighted imaging
ECASS: European Cooperative Acute Stroke Study
IQR: interquartile range
IST-3: Third International Stroke Trial
MCA: middle cerebral artery
NIHSS: NIH Stroke Scale
OR: odds ratio
